# Immediate risk of myocardial infarction following physical exertion, tea, and coffee: A case-crossover study in Thailand

**DOI:** 10.1371/journal.pone.0210959

**Published:** 2019-01-17

**Authors:** Vitool Lohsoonthorn, Thanapoom Rattananupong, Keona Wynne, Colbren Thomas, Harpreet S. Chahal, Hanna Y. Berhane, Elizabeth Mostofsky, Nudsinee Wuttithai, Bizu Gelaye

**Affiliations:** 1 Faculty of Medicine, Chulalongkorn University, Bangkok, Thailand; 2 Department of Epidemiology, Harvard T. H. Chan School of Public Health, Boston, Massachusetts, United States of America; 3 Mississauga Academy of Medicine, University of Toronto Mississauga, Mississauga, Canada; 4 Addis Continental Institute of Public Health, Addis Ababa, Ethiopia; 5 Chiangrai Prachanukroh Hospital, Chiangrai, Thailand; University of Hawai’i at Manoa College of Tropical Agriculture and Human Resources, UNITED STATES

## Abstract

**Background:**

Physical exertion and caffeine consumption are associated with acute myocardial infarction (MI). However, physical exertion and caffeine consumption have not been examined as immediate triggers of MI in low and middle-income countries.

**Objective:**

Using a self-matched case-crossover design, we examined the acute risk of MI in the hour following episodes of physical exertion, caffeinated coffee, and tea consumption among MI survivors in Thailand.

**Methods:**

A total of 506 Thai participants (women = 191, men = 315) were interviewed between 2014 and 2017 after sustaining an acute MI. We compared each subject’s exposure to physical exertion and consumption of caffeine- containing beverages in the hour preceding the onset of MI with the subject’s expected usual frequency in the prior year to calculate relative risks (RRs) and 95% confidence intervals (95%CIs).

**Results:**

Of the 506 participants, 47 (9.3%) engaged in moderate or heavy physical exertion, 6 (1.2%) consumed tea, and 21 (4.2%) consumed coffee within the hour before MI. The relative risk of MI after moderate or heavy physical exertion was 3.0 (95% CI 2.2–4.2) compared to periods of no exertion, with a higher risk among more sedentary participants compared to active participants. Compared to times with no caffeinated beverage consumption, there was a higher risk of MI in the hour following consumption of caffeinated tea (RR = 3.7; 95%CI: 1.5–9.3) and coffee (RR = 2.3; 95%CI: 1.4–3.6).

**Conclusion:**

Physical exertion, coffee and tea consumption were associated with a higher risk of MI in the subsequent hour compared to times when the participants were sedentary or did not consume caffeinated beverages. Our study identifies high-risk populations for targeted screening and intervention to prevent acute MI.

## Introduction

Cardiovascular disease (CVD) contributes to 30% of global mortality [1]. According to estimates by the World Health Organization, 80% of premature death due to CVD occurs in low and middle-income countries [1–3]. In Southeast Asia, CVD has emerged as a leading cause of mortality. For example, rates of hospitalized MI patients increased from 80.70 per 100,000 people in 2009 to 96.68 per 100,000 in 2011 [4]. Recent increases in CVDs are attributed to population growth, aging, unhealthy diets, increased alcohol consumption, lack of physical activity, weight gain, and exposure to stress [1]. Epidemiological evidences also shows that short-term exposures trigger myocardial infarction (MI) including physical exertion [5,6], caffeine consumption [7], sexual activity [8], anger or emotional upset [9,10]. However, many of these previous studies were conducted in occidental countries [5,6,9–11].

Given the scarcity of epidemiologic studies evaluating acute risk factors for MI in Southeast Asia, we conducted this study to assess whether physical exertion, caffeinated coffee, and tea consumption are acute triggers of nonfatal acute MI in the subsequent hour among patients in Thailand.

## Methods

### Study population

Study subjects were Thai men and women who participated in the Stroke and Myocardial Infarction Acute Risk Factors in Thailand (SMART) study between October 2014 and March 2017. A total of 506 participants were interviewed at King Chulalongkorn Memorial, Lampang, Chiangrai Prachanukroh, and Sunpasitthiprasong hospitals in Thailand after sustaining an acute MI. Trained research personnel conducted detailed chart reviews and structured patient interviews. The eligibility criteria used were: at least one creatine kinase level above the upper limit of normal for the clinical laboratory performing the test, positive creatinine kinase-MB isoenzymes, an identifiable onset of pain or other symptoms typical of infarction, and the ability to complete a structured interview.

### Study design

The case-crossover design was selected to examine the transient effect of intermittent exposures to caffeine consumption and exercise on MI risk [12,13]. Rather than comparing different people at the same time, the case-crossover design compares the same person at different times. As a result, there is no confounding by fixed or slowly varying characteristics such as sex, age, and prior medical history. This study design involves collecting information on exposure (e.g., participation in physical exertion) immediately preceding the event (e.g., MI) and comparing this with the expected frequency of exposure over a similar time period based on the study patient’s habitual pattern.

### Data collection

MI patients coming to the health facility were consecutively invited to participate in the study.

Data were collected on patient demographics and putative risk factors for MI. A structured interview identified the time, place, and intensity of MI pain and other symptoms, as well as the timing and estimated usual frequency of exposure to potential triggers of MI onset during the prior year, including physical exertion, caffeinated coffee and tea consumption. Participants who reported exposure to potential triggers in the prior year reported their most recent exposure prior to MI onset with the following response options: never, at the time of event onset, ½ hour before, 1 hour before, 2 hours before, 3–6 hours before, 6–24 hours before, 1–2 days before, 3–4 days before, or ≥ 5 days before. All patient interviews, as well as chart reviews, were conducted by trained research personnel.

### Statistical analysis

A self-matched case crossover analysis was conducted to compare each participants’ exposures in the hour prior to the MI with the same individual’s usual exposure. Using this approach individuals are compared to themselves at other times, so there is no confounding by sex or age. We used methods for sparse data [14], to calculate Mantel-Haenszel relative rate ratios (RR) for person-time and 95% confidence intervals (CIs). The details of this approach have been previously described [15–17]. Briefly, in the case-crossover design, data are stratified on each individual event. We multiplied the usual annual frequency of exposure (to moderate or heavy physical exertion, tea, or coffee) by the hypothesized window of its physiologic effect (1 hour) to calculate annual exposure time. We then subtracted this value from total hours in a year to calculate annual non-exposure time.

We examined whether the immediate risk of MI following moderate or heavy physical exertion or following coffee or tea consumption was different according to strata of sex, age (<65, 65+ years), and smoking status (never, current) using a Wald χ2 test of homogeneity [14]. We also tested whether the association for moderate or heavy physical exertion was different between people who habitually engaged in physical exertion more than 5 times per week compared to those who engaged in physical exertion fewer than 5 times per week.

We conducted a sensitivity analysis excluding study participants who reported the following exposures in the hour preceding their MI: alcohol, smoking, other caffeinated beverages, or moderate or heavy physical exertion depending on the association tested. In another sensitivity analysis, we used the usual frequency of exposure based on the week before MI as the comparator. All reported *P*-values are 2-sided, and statistical significance is set at 0.05.

### Ethical issues

Written informed consent was obtained from all study participants. All study procedures were approved by the institutional review boards of the participating hospitals and Faculty of Medicine, Chulalongkorn University, Thailand and the Office of Human Research Administration, Harvard T.H. Chan School of Public Health, Boston, USA.

## Results

As shown in Table 1, most participants were between the ages of 55–74 years (mean age: women: 65.5 ± 11.8 years vs. men: 61.5 ± 11.8 years) with a primary school education. Men were more likely to be overweight or obese as compared with women. Current smoking was reported by 42.5% of men and 13.1% of women. More than half of participants had hypertension and one-fourth were diabetic. Men were more likely to suffer from a ST-elevation Myocardial Infarction (STEMI) than women (66.7% vs. 55.6%; Table 1).

**Table 1:** Characteristics of patients with myocardial infarction in Thailand (N = 506).

Characteristics	Women (N = 191) n (%)	Men (N = 315) n (%)
Age, mean ± SD	65.5 ± 11.8	61.5 ± 11.8
Age		
<55	35 (18.3)	91 (28.9)
55–64	55 (28.8)	103 (32.7)
65–74	57 (29.8)	70 (22.2)
75+	44 (23.0)	51 (16.2)
Education		
Less than primary school	61 (31.9)	40 (12.7)
Primary school completed	105 (55.0)	170 (54.0)
Secondary or high school completed	7 (3.7)	50 (15.9)
College/university or postgraduate degree	18 (9.4)	55 (17.5)
BMI (kg/m^2^) *		
<18.5	44 (24.9)	32 (10.6)
18.5–24.9	92 (52.0)	166 (55.0)
25–29.9	31 (17.5)	80 (26.5)
≥30	10 (5.6)	24 (7.9)
Smoking status		
Never	141 (73.8)	129 (41.0)
Former	25 (13.1)	52 (16.5)
Current	25 (13.1)	134 (42.5)
Prior stroke	3 (1.6)	5 (1.6)
Diabetes	51 (26.7)	73 (23.2)
Hypertension	127 (66.5)	168 (53.3)
Atrial fibrillation	4 (2.1)	6 (1.9)
Hypercholesterolemia	45 (23.6)	77 (24.4)
Type of myocardial infarction*		
STEMI	105 (55.6)	210 (66.7)
Non-STEMI	84 (44.4)	105 (33.3)

*Due to missing data, frequencies may not sum to group total.

Abbreviations: SD, standard deviation; STEMI, ST-elevation Myocardial Infarction; Non-STEMI, non-ST-elevation Myocardial Infarction

### Physical exertion

Of the 506 participants with MI, 270 (53%) reported that they participated in moderate or heavy physical exertion in the past year. Eight participants provided no information on usual exertion so were excluded from analysis. Among 262 participants who engaged in moderate or heavy exertion in the past year, 162 (61.8%) reported daily exertion, 83 (31.7%) 1 or more times per week, and 17 (6.5%) reported exertion 1 or more times per month. The median frequency of physical exertion was 7 times per week, and the average duration of each episode was 52 minutes (interquartile range, 18–53).

Among 262 participants who engaged in moderate or heavy physical exertion in the prior year, 47 of them reported participating in moderate or heavy physical exertion one hour prior to onset of MI. The risk of MI was 3-fold higher (RR = 3.0; 95% CI: 2.2–4.2) within an hour of moderate or heavy physical exertion compared to other times (Table 2). The RR of MI was 2.8-fold higher (95% CI: 2.0–4.0) for those who habitually engaged in physical exertion more than 5 times per week, and it was 5.0-fold higher (95% CI: 2.4–10.3, *P*-homogeneity = 0.17) for those who engaged in physical exertion 5 or fewer times a week. The RR of MI following moderate or heavy physical exertion was lower for men (RR = 2.5, 95% CI: 1.7–3.6) than for women (RR = 5.5, 95% CI: 3.0–10.3, *P*-homogeneity = 0.03), higher for participants 65 and older (RR = 6.3, 95% CI: 3.8–10.5) than for those < 65 years old (RR = 2.1, 95% CI: 1.3–3.2, *P*-homogeneity < 0.001), and higher for never-smokers (RR = 4.4, 95% CI: 2.8–6.8) than for current-smokers (RR = 2.1, 95% CI: 1.2–3.5, *P-*homogeneity = 0.02) (Table 2). Never-smokers were, on average, 9.5 years older than current smokers.

**Table 2:** Relative risk of myocardial infarction within an hour of moderate or heavy physical exertion.

	Number exposed in the past year	Number exposed in the past hour	Relative risk (95% CI)	*P* for homogeneity
All	262	47	3.0 (2.2–4.2)	
Habitual exertion (times/week)		8	5.0 (2.4–10.3)	0.17
≤ 5		39	2.8 (2.0–4.0)	
> 5				
Sex				
Male		31	2.5 (1.7–3.6)	0.03
Female		16	5.5 (3.0–10.3)	
Age				
< 65 years old		26	2.1 (1.3–3.2)	<0.001
≥ 65 years old		21	6.3 (3.8–10.5)	
Smoking				
Never-smoker		26	4.4 (2.8–6.8)	0.02
Current smoker		16	2.1 (1.2–3.5)	
No co-exposure during hour prior to MI onset*		32	2.7 (1.4–3.6)	

* Excluding participants who reported the following exposures in the hour prior to MI onset: caffeinated beverages, alcoholic beverages, cigarettes

### Coffee and tea

Caffeinated tea consumption in the past year was reported among 68 (13.4%) participants. Of these, four patients provided no information on usual tea consumption and were excluded from final analyses. Among 64 participants who consumed tea in the past year, 30 (46.9%) consumed at least 1 serving of tea per day, 13 (20.3%) reported consuming tea at least once per week, 14 (21.9%) at least once per month, and 7 (10.9%) less than once per month. The median frequency of tea consumption among those who consumed tea was 3 times per week. Of 506 patients, 180 (35.6%) reported that they consumed caffeinated coffee in the prior year. However, 3 patients were excluded from final analyses due to missing information on usual coffee consumption.

Among the 177 patients who reported coffee consumption in the past year, 21 patients consumed at least 1 serving of coffee within an hour of MI onset. The risk of MI was 2.3-fold higher within an hour of caffeinated coffee consumption compared to periods of non-use (95%CI: 1.4–3.6) (Table 3). The RRs for caffeinated coffee consumption did not vary by sex (p = 0.77) or age (p = 0.14) but did vary by smoking status. Compared to patients who did not consume coffee, the RR for MI after coffee consumption among never-smokers was higher (RR = 3.4, 95% CI: 2.8–6.8) compared with current-smokers (RR = 1.0, 95% CI: 0.4–2.5, *P*-value for homogeneity = 0.03). Never-smokers were also, on average, 9.5 years older than current smokers.

**Table 3:** Relative risk of myocardial infarction in the hour following caffeinated coffee consumption.

	Number exposed in the past year	Number exposed in the past hour	Relative risk (95% CI)	*P* for homogeneity
All	177	21	2.3 (1.4–3.6)	
Sex				
Male		16	2.2 (1.3–3.7)	0.77
Female		5	2.6 (1.0–6.7)	
Age				
< 65 years old		13	1.8 (1.0–3.3)	0.14
≥ 65 years old		8	3.8 (1.7–8.2)	
Smoking				
Never-smoker		11	3.4 (2.8–6.8)	0.03
Current smoker		5	1.00 (0.4–2.5)	
No co-exposure during hour prior to MI onset*		14	2.1 (1.2–3.6)	

* Excluding participants who reported the following exposures in the hour prior to MI onset: moderate or vigorous physical exertion, caffeinated tea, alcoholic beverages, cigarettes

Among the 64 patients who consumed tea in the past year, 6 participants consumed at least 1 serving of caffeinated tea within an hour of MI onset. The immediate risk of MI onset was 3.7-fold higher (95% CI: 1.5–9.3) within an hour of caffeinated tea consumption compared to periods of non-use (Table 4).

**Table 4:** Relative risk of myocardial infarction in the hour following caffeinated tea consumption.

	Number exposed in the past year	Number exposed in the past hour	Relative risk (95% CI)
All	64	6	3.7 (1.5–9.3)
No co-exposure during hour prior to MI onset*		5	4.6 (1.7–12.8)

* Excluding participants who reported the following exposures in the hour prior to MI onset: moderate or vigorous physical exertion, caffeinated coffee, alcoholic beverages, cigarettes.

### Sensitivity analyses

The RR of MI in the hour after moderate or heavy physical exertion, tea, and coffee remained elevated when we excluded participants who were exposed to other potential triggers in the hour before MI (e.g., other caffeinated beverages, alcoholic beverages, cigarettes, moderate or heavy physical exertion). Additionally, the RR of MI in the hour after moderate or heavy physical exertion, tea, and coffee consumption also did not change materially when we compared recent exposure to exposure in the week before MI rather than exposure in the prior year.

## Discussion

Moderate and heavy physical exertion, caffeinated coffee and caffeinated tea consumption were associated with a higher risk of MI in the subsequent hour after exposure. The frequency of habitual physical exertion modified the relative risk of MI with less active participants having a higher risk of MI in the hour immediately following physical exertion compared to more active participants, although this difference did not reach statistical significance. Consumption of caffeinated coffee and tea was also associated with a higher risk of MI in the subsequent hour.

Our results for physical exertion are consistent with those from previous case- crossover studies [5,6,8]. In one of the first case-crossover studies, Mittleman *et al*. found an increased risk of MI in the hour immediately following heavy physical exertion in a US cohort (RR = 5.9; 95% CI, 4.6–7.7) as compared to periods of lower physical exertion or rest [5]. The Swedish Onset Study, a part of the Stockholm Heart Epidemiology Program (SHEEP), found a 6.1-fold increased RR (95% CI, 4.2–9.0) in the hour following strenuous physical exertion compared to periods of lower exertion or rest [6]. In Costa Rica, Baylin *et al*. found a RR of 4.9 (95% CI 3.7–6.5) in the hour following physical exertion [8]. The results of our study and previous research demonstrate that the transiently increased risk of MI following isolated episodes of heavy physical exertion is of potential concern. Of note, our results and those of others [8], emphasize the importance of habitual physical exertion in preventing MI risk [18]. The plausible biological mechanisms for the triggering effect of physical exertion include increased sympathetic activity, platelet aggregability and coronary vasomotor tone, particularly in individuals without habitual physical activity[19].

Our findings also show an increased risk of MI in the hour following coffee and tea consumption. To the best our knowledge, our study is the first to examine the acute risk of MI in relation to caffeine-containing tea consumption using a case-crossover design. Therefore, our results can be tentatively compare with previous studies that investigated the risk of MI following coffee or alcohol consumption. Baylin *et al*. in Costa Rica found a 1.49-fold increased risk of acute MI in the hour following coffee consumption (RR = 1.49; 95% CI 1.17–1.89) [7]. Similarly, our results show a higher risk of MI within the hour following coffee consumption (RR = 2.3; 95% CI, 1.4–3.6) or tea consumption (RR = 3.7; 95% CI: 1.5–9.3). In a recent meta-analysis, alcohol consumption was shown to have an increased risk of cardiovascular events in the hour after consumption. However, this risk was attenuated by 24 hours and protective after one week [20]. The increased risk MI in the hour following coffee and tea consumption may also be protective long-term on cardiovascular outcomes.

The observed association of coffee and tea consumption with MI risk may be a result of the chemical composition of these beverages. Caffeine is a widely used psychoactive substance that stimulates the central nervous system [21]. A United States Food and Drug Administration (USFDA) report suggests one serving of coffee contains approximately 50–330 mg of caffeine; while in a serving of black tea, there is approximately 40-74mg [22]. However, caffeine content in these beverages is highly variable [22–24]. Coffee consumption has been shown to have significant effects on the cardiovascular system, carbohydrate and lipid metabolism, and mortality reduction [25,26]. However, there is little evidence of effects on arrhythmia or coronary heart disease [25,27,28].

Other compounds may contribute to the biological effects of coffee or tea consumption. For example, flavonoids found in black tea may reduce the risk of MI by inhibiting low density lipoprotein cholesterol oxidation, reducing platelet aggregation, or reducing ischemic damage, and improving endothelial function in an additive fashion [29,30]. Studies also show that flavonoids inhibit LDL oxidation, possibly reducing macrophage superoxide production [31,32]. It has been proposed that flavonoids inhibit platelet aggregation by the suppression of phosphodiesterase or cyclooxygenase activity, yet this hypothesis remains unclear *in vitro* [32,33]. While alone, coffee exhibits adverse effects on the cardiovascular system such as serum cholesterol, blood pressure, and plasma homocysteine, caffeine is the only major compound present in filtered coffee thus, the important biological effects of flavonoids in tea cannot be ignored [21]. The differing chemical molecules or caffeine content found in tea and coffee could account for the observed odds ratios.

As with case-crossover studies, confounding by fixed and slow-varying characteristics were eliminated due to the self-matching design of the study. However, our study has a few limitations. First, the study does not account for characteristics that may change over time. For example, cardiovascular events follow a circadian peak, with the greatest risk in the morning [34,35]. Second, bias by time may affect the results of coffee and tea consumption, as caffeinated drinks are mostly consumed in the morning. Third, there may be misclassification of the usual frequency of physical exertion and tea or coffee consumption. Subjects were interviewed and asked to report their usual frequency during the past year, opening the study up to recall bias and social desirability bias. We attempted to minimize recall bias by using a standardized questionnaire and providing participants with several options for when they were last exposed to a potential trigger so as to not inform them of a hypothesized hazard period. Future studies should aim to examine the types of exercise the participants engage in and investigate further how much lifestyle impacts physical exertion in relation to MI. Also, types of coffee and tea consumed should be documented in order to compare the average amount of caffeine consumed. Lastly, our study sample included relatively healthy MI patients. As such, our results may not be generalizable to patients who experience severe or fatal MI.

## Conclusions

In our study of Thai population, we found an acute increased risk of MI in the hour following episodes of moderate or heavy physical exertion, and consumption of caffeinated coffee and tea. The association with episodes of physical exertion was higher among participants who were habitually sedentary, although the difference did not reach statistical significance. Future studies should aim to examine whether particular types of physical exertion are associated with heightened MI risk and, similarly, whether specific types of caffeinated coffees and teas are particularly deleterious.
